# Perceptions of the determinants of health across income and urbanicity levels in eight countries

**DOI:** 10.1038/s43856-024-00493-z

**Published:** 2024-06-06

**Authors:** Salma M. Abdalla, Ethan Assefa, Samuel B. Rosenberg, Mark Hernandez, Shaffi Fazaludeen Koya, Sandro Galea

**Affiliations:** 1 Department of Global Health, Boston University School of Public Health, Boston, MA USA; 2 Department of Epidemiology, Boston University School of Public Health, Boston, MA USA; 3 Boston University School of Public Health, Boston, MA USA; 4 Urban Health Collaborative, Drexel University Dornsife School of Public Health, Philadelphia, PA USA

**Keywords:** Public health, Disease prevention

## Abstract

**Background:**

A clear understanding of public perceptions of the social determinants of health remains lacking. This paper aimed to describe the relationship between income and urbanicity levels and public views of the determinants of health in eight middle-and high-income countries that varied across multiple characteristics.

**Methods:**

We conducted a cross-sectional online survey in Brazil, China, Germany, Egypt, India, Indonesia, Nigeria, and the United States. Respondents were asked to select what they considered to be most important for health out of a list of ten determinants. We stratified the results by income and urbanicity levels and tested significance of differences between groups using two-tailed χ^2^ tests. Multivariable logistic regression models tested associations between demographic factors and the likelihood of respondents selecting the genetics, healthcare, income and wealth, or social support determinants.

**Results:**

Here we show 8753 respondents across eight countries. Rankings of determinants are similar across income groups, except for two determinants. Respondents in the highest income group rank genetics in higher proportions (32.4%, 95%CI: 29.0%,35.8%) compared to other income groups. Conversely, those in lowest income group rank social support more frequently (27.9%, 95%CI: 25.3%,30.7%) than other income groups.

Those living in urban settings rank healthcare in higher proportions (61.2%, 95%CI: 59.0%,63.4%) compared to non-urban respondents; meanwhile, higher proportions (26.6%, 95%CI: 24.9%,28.3%) of non-urban respondents rank social support as important for health compared to urban respondents.

**Conclusion:**

Demographic factors play a role in shaping public views of what affects health. Advancing public understanding about determinants of health requires tailoring public health messaging to account for socioeconomic position within a population.

## Plain language summary

Various factors determine our health, such as our environment, education, and genetics. Here, we explore how people from different income and urban backgrounds view the factors that affect health. We surveyed participants from Brazil, China, Germany, Egypt, India, Indonesia, Nigeria, and the United States. Respondents selected the most important factors for health from a list of ten choices. Overall, people’s views on these factors were comparable across income groups, with exceptions. Those with higher incomes tended to emphasize the importance of genetics more, while lower-income individuals valued social support. People in urban areas prioritized healthcare, while those in non-urban areas valued social support. These findings suggest that factors such as income and where people live influence their views about health determinants. These factors should be considered in public health messaging and strategies to promote health amongst different groups.

## Abstract (web summary)

Abdalla et al. examine how demographic factors shape perceptions of what matters for health across eight countries. They show that high income groups rank genetics as important, urban groups rank healthcare as important, and both low-income groups and non-urban groups rank social support as important.

## Introduction

The last quarter of the 20th century saw a substantial increase in scholarship that focused on the interactions between people, their environments, and health^1,2^. This led to a growing understanding of the social and economic forces that shape health at both the individual and population level—often referred to as the social determinants of health (SDoH)^3–8^.

The emergence of scholarship around SDoH has been accompanied by a growing interest among decision-makers in the forces that affect health beyond medical care^9–13^. Initiatives such as the Health in All Policies approach have aimed to establish a pipeline from scholarship to action on SDoH. However, these efforts have yet to translate into comprehensive policies and political action on health shaped by an SDoH framing^14–18^.

Political action often requires widespread public consensus. Thus, buy-in from the public that investment in SDoH is central to establish healthy populations is indispensable to catalyzing action. A well-informed public can  help hold decision-makers accountable  for resource allocation choices that affect health outcomes^19^.

Identities and demographic characteristics often shape worldviews, which, in turn, can affect political engagement and action^20–27^. There is some evidence that demographic factors also contribute to perceptions around what shapes health^28,29^. Emerging literature has shown the role of income level in shaping views on health on a national level. For example, one analysis of 29 countries showed that a higher average income for a country correlated with greater likelihood of selecting genetics as an important determinant of health^17^. This is consistent with an analysis of eight countries, which found that respondents in high-income countries (HICs) ranked genetics as the most important determinant of health in higher frequency compared to respondents from middle-income countries^30^. While not particularly focused on health, the literature also shows that people residing in urban settings often have different views compared to those living in non-urban settings^31,32^.

Given the importance of income and urbanicity in shaping cultural conversations, a better understanding of how both influence perceptions of health determinants can provide insights into designing effective communication around SDoH. Yet, there is little research that aims to understand public views about what matters for health, particularly stratified by socio-demographic characteristics. This paper aims to add to the literature through assessing the relationship between income and urbanicity and perceptions of important determinants of health in eight middle- and high-income countries.

A higher proportion of high income respondents rank genetics as the most important determinant of health, compared to respondents in other income groups. Respondents in the lowest income group rank social support as the most important for health compared to respondents in other income groups. Respondents living in urban settings more often rank healthcare as important compared to non-urban groups, while respondents living in non-urban settings more often rank social support as the most important compared to urban groups.

## Methods

We conducted a cross-sectional, anonymous, online survey in eight nations between September 16th, 2020, and November 1st, 2020. The countries were in different geographic regions and varied substantively in their gross-income per capita (GDP), language, religion, and demographic composition.

We used RIWI— the global platform for trend-tracking and prediction which utilizes Random Domain Technology™ (RDIT™)—for our study. RDIT™ has been used in multiple global surveys by international organizations^33^. This research was exempted from a comprehensive ethical review by the Institutional Review Board (IRB) at Boston University (IRB Number: H-40806) due to data anonymity.

### Study population and sample size

The study population involved adult (18+ years) internet users in Brazil, China, Germany, Egypt, India, Indonesia, Nigeria, and the United States. Users who made errors while typing website addresses or inadvertently navigated to a domain or subdomain owned or controlled by RIWI were redirected to one of thousands of RIWI URLs. A random sample of these users was then invited to participate in a survey using a series of algorithms. Respondents who opted-in received the survey in the language predominantly used in their country (e.g., Portuguese in Brazil and Arabic in Egypt). They provided consent at the start of the survey and were only allowed to respond once based on their IP address. The technology implemented various filters to prevent non-human respondents or ‘bots’ from responding. None of the respondents were compensated and the platform also utilized an algorithm to exclude paid panel respondents.

We established a sample size of 1000 or more responses per country. The survey was seen by 37,566 individuals and 8,753 chose to fill out the full survey, garnering a response rate of 23.3%. The remainder of people exposed to the survey either skipped or closed the page without submitting the survey.

### Demographic variables

Demographic data collected included country of residence, age, area of residence (or place of living), education level, and income level. Area of residence was defined as large city, suburb, small town/village, and rural area/farm. We aggregated the area of residence variable responses into an urban/non-urban dichotomy in this analysis. Those who selected suburb, small town/village, or rural area/farm as their area of residence were categorized as non-urban residents, and those who chose large city were classified as urban residents. Education level was assessed with the following categories: less than secondary/no formal education, completed secondary school, and college/university degree or more. Income was stratified into brackets (highest income, upper-middle income, middle income, lower-middle income, and lowest income) based on respondents’ reported monthly household income in the currency of their country of residence. Monthly household income groups by country are provided in Supplementary Table 1.

### Determinants of health variables

We ascertained a list of ten determinants of health based on existing literature and established frameworks for key determinants of health. The ten determinants provided to respondents were healthcare, education, built environment (e.g., housing or neighborhood conditions), employment conditions, income & wealth, genetics, childhood conditions, culture, social support, and politics.

Respondents were asked to choose which determinant they considered as important for health. Then, they were asked to select again from the remaining pool of choices two more times to provide their top three choices for determinants of health. These top three were aggregated into a composite measure of respondents’ first, second, and third choice for key determinants of health. This composite measure was examined, in addition to their first choice, in the analyses of the data.

### Statistical analysis plan

First, we summarized the unweighted frequencies and age- and gender-weighted percentages of income levels and urbanicity in the overall sample and by country. Second, we conducted bivariable descriptive analyses of percentages with 95% confidence intervals (CIs) with Rao-Scott correction looking at the percentage of respondents who considered each determinant as one of the three most important to health. We stratified results by income level and urbanicity status for the overall sample and by country. We used a two-tailed chi-square test for significance testing between groups.

Third, we conducted four multivariable logistic regressions that separately assessed genetics, healthcare, income and wealth, or social support as one of the three most important determinants for health. The predictors for all models consisted of all demographic variables: country, gender, age, income group, education, and area of residence by urbanicity. We weighted all tables, regression models, and figures by gender and age. We conducted all analyses twice—first, for the composite metric that combined the first, second, or third choices, and second, only for the first choice of health determinant.

A *p* < 0.05 was considered statistically significant throughout the analysis. The statistical programming software, R (version 4.1.2), was used for data cleaning, analysis, and visualization; we used the “tidyverse”, “data.table”, “survey”, “srvyr”, “gt”, and “gtsummary” packages for our analysis^1–5^. We adhered to the directives set by the Strengthening the Reporting of Observational Studies in Epidemiology (STROBE) initiative for cross-sectional studies.

### Reporting summary

Further information on research design is available in the Nature Portfolio Reporting Summary linked to this article.

## Results

### Demographic characteristics

A total of 8753 distinct users answered the survey. There was a comparable distribution of responses between countries, China had the highest response (*N* = 1282) and Nigeria had the lowest response (*N* = 1014). The full demographic characteristics of the sample are available in a prior publication (Supplementary Table 2)^30^.

Income level was relatively evenly distributed among countries. Nigeria had the widest response gap between lowest income (28.1%, 95% CI: 25.0%, 31.3%) and highest income (11.4%, 95% CI: 9.0%, 14.1%) respondents and Brazil had the narrowest response gap between lowest income (21.9%, 95% CI: 18.9%, 25.1%) and highest income (24.6%, 95% CI: 21.2%, 28.3%) respondents. The proportion of those living in non-urban areas ranged from 42.4% (95% CI: 38.8%, 46.1%) in Brazil to 70.0% (95% CI: 66.2%, 73.7%) in Germany. China, Germany, India, Indonesia, and the United States contained higher non-urban proportions of respondents, while Brazil, Egypt and Nigeria contained higher urban proportions (Table 1).

**Table 1:** Income, and urbanicity, in an eight-country survey

	Overall, *N* = 8753		Brazil, *N* = 1075		China, *N* = 1282		Egypt, *N* = 1082		Germany, *N* = 1036		India, *N* = 1173		Indonesia, *N* = 1026		Nigeria, *N* = 1014		United States, *N* = 1065	
Characteristic	*n*	% [95% CI]	*n*	% [95% CI]	*n*	% [95% CI]	*n*	% [95% CI]	*n*	% [95% CI]	*n*	% [95% CI]	*n*	% [95% CI]	*n*	% [95% CI]	*n*	% [95% CI]
Income group																		
Highest income	1404	20.4% [19.1%, 21.7%]	183	24.6% [21.2%, 28.3%]	302	26.3% [21.5%, 31.5%]	119	13.8% [11.0%, 16.9%]	141	14.8% [12.0%, 17.9%]	138	16.7% [13.5%, 20.3%]	158	24.5% [20.1%, 29.3%]	88	11.4% [9.0%, 14.1%]	275	29.2% [26.2%, 32.4%]
Upper-middle income	999	13.8% [12.7%, 14.9%]	169	18.0% [15.1%, 21.1%]	187	18.0% [14.1%, 22.4%]	126	13.8% [11.1%, 16.7%]	90	9.9% [7.6%, 12.7%]	103	11.0% [8.5%, 13.8%]	111	16.0% [12.0%, 20.6%]	102	12.8% [10.4%, 15.5%]	111	11.0% [9.0%, 13.1%]
Middle income	1524	20.1% [18.9%, 21.4%]	186	19.3% [16.5%, 22.4%]	287	25.9% [21.1%, 31.0%]	253	27.2% [23.7%, 30.8%]	141	16.0% [13.1%, 19.3%]	224	25.3% [21.5%, 29.3%]	142	15.5% [12.3%, 19.2%]	160	18.2% [15.4%, 21.2%]	131	13.9% [11.7%, 16.4%]
Lower-middle income	1588	20.9% [19.8%, 22.2%]	162	16.2% [13.5%, 19.2%]	134	13.5% [10.0%, 17.6%]	205	21.6% [18.5%, 24.9%]	256	29.6% [25.8%, 33.6%]	204	21.7% [18.2%, 25.6%]	146	13.8% [10.9%, 17.1%]	269	29.6% [26.3%, 33.0%]	212	22.0% [19.3%, 24.8%]
Lowest income	2115	24.8% [23.5%, 26.1%]	220	21.9% [18.9%, 25.1%]	180	16.3% [12.2%, 21.0%]	237	23.7% [20.5%, 27.1%]	290	29.6% [25.9%, 33.5%]	293	25.3% [21.8%, 29.1%]	353	30.2% [26.0%, 34.6%]	298	28.1% [25.0%, 31.3%]	244	23.9% [21.2%, 26.8%]
Area of residence by urbanicity																		
Urban	3500	43.2% [41.7%, 44.7%]	539	57.6% [53.9%, 61.2%]	565	44.8% [39.4%, 50.3%]	512	53.2% [49.4%, 57.0%]	288	30.0% [26.3%, 33.8%]	417	40.8% [36.7%, 44.9%]	332	35.1% [30.8%, 39.7%]	507	53.2% [49.7%, 56.7%]	340	32.1% [29.1%, 35.2%]
Non-Urban	4808	56.8% [55.3%, 58.3%]	468	42.4% [38.8%, 46.1%]	633	55.2% [49.7%, 60.6%]	510	46.8% [43.0%, 50.6%]	699	70.0% [66.2%, 73.7%]	666	59.2% [55.1%, 63.3%]	656	64.9% [60.3%, 69.2%]	482	46.8% [43.3%, 50.3%]	694	67.9% [64.8%, 70.9%]

Responses with missing data on income (1123) and area of residence by urbanicity (445) were removed (*N* = 7630). Percentages were weighted to adjust for age and gender. Percentages may not sum to 100 due rounding.

### Ranking of determinants by income group

**Fig. 1:**
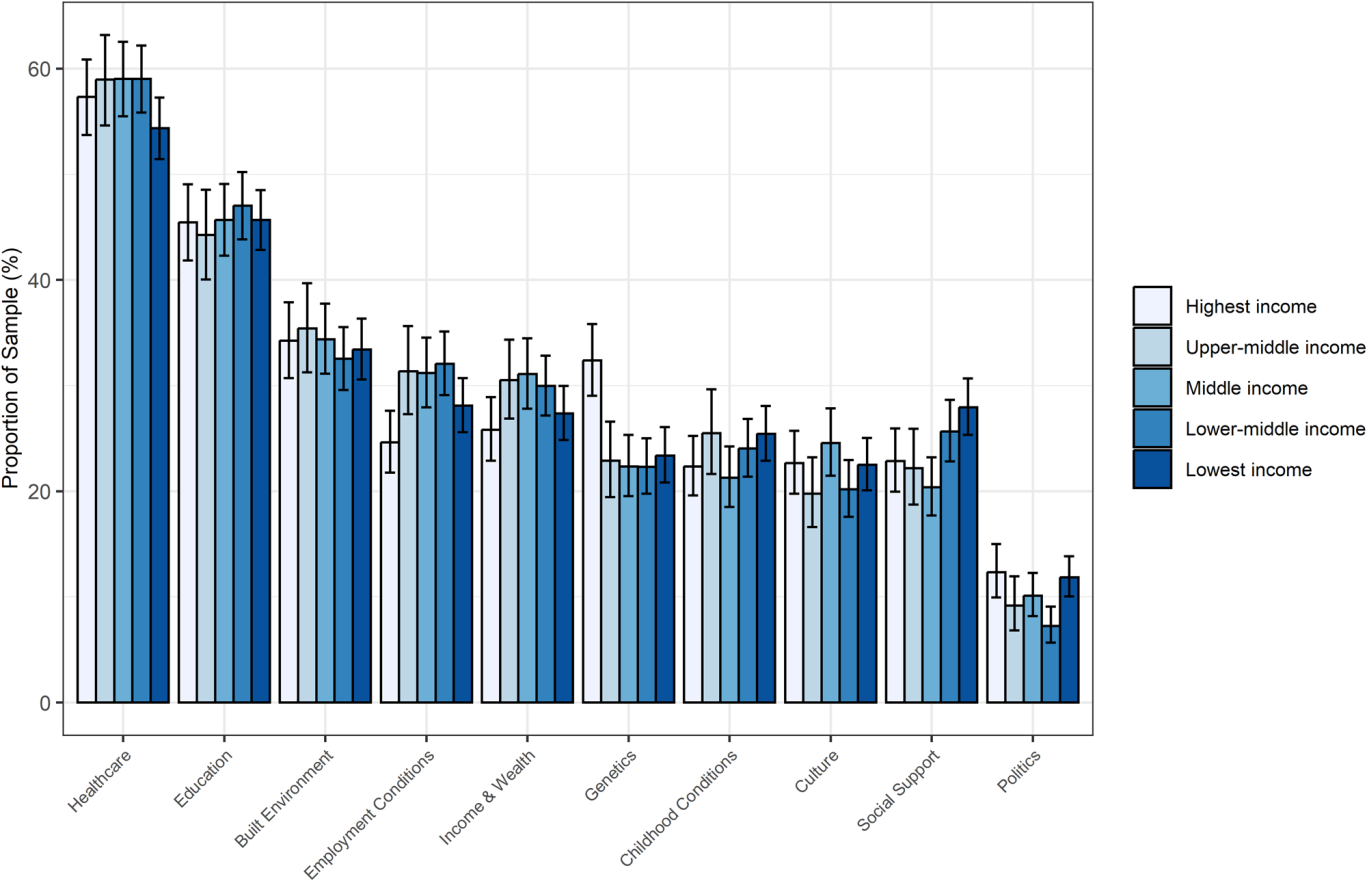
Rankings of what respondents think matters for health, stratified by income level. All responses with missing data for income levels (1123) were excluded (*N* = 7630). Results were weighted to adjust for age and gender. The rankings reflect a composite of the first, second, or third choice for important health determinants for health. Error bars represent the 95% confidence interval.

Figure 1 shows that, using the composite metric, rankings were generally similar by income group. Overall, the highest income and the lowest income showed comparable ranking trends. This was particularly evident in the ranking of politics in both the overall sample and within countries. In the overall sample, 12.3% (95% CI: 10.0%, 15.0%) and 11.9% (95% CI: 10.0%, 13.8%) of respondents ranked politics as one of the top three most important determinants of health in the highest income and lowest income groups, respectively.

**Fig. 2:**
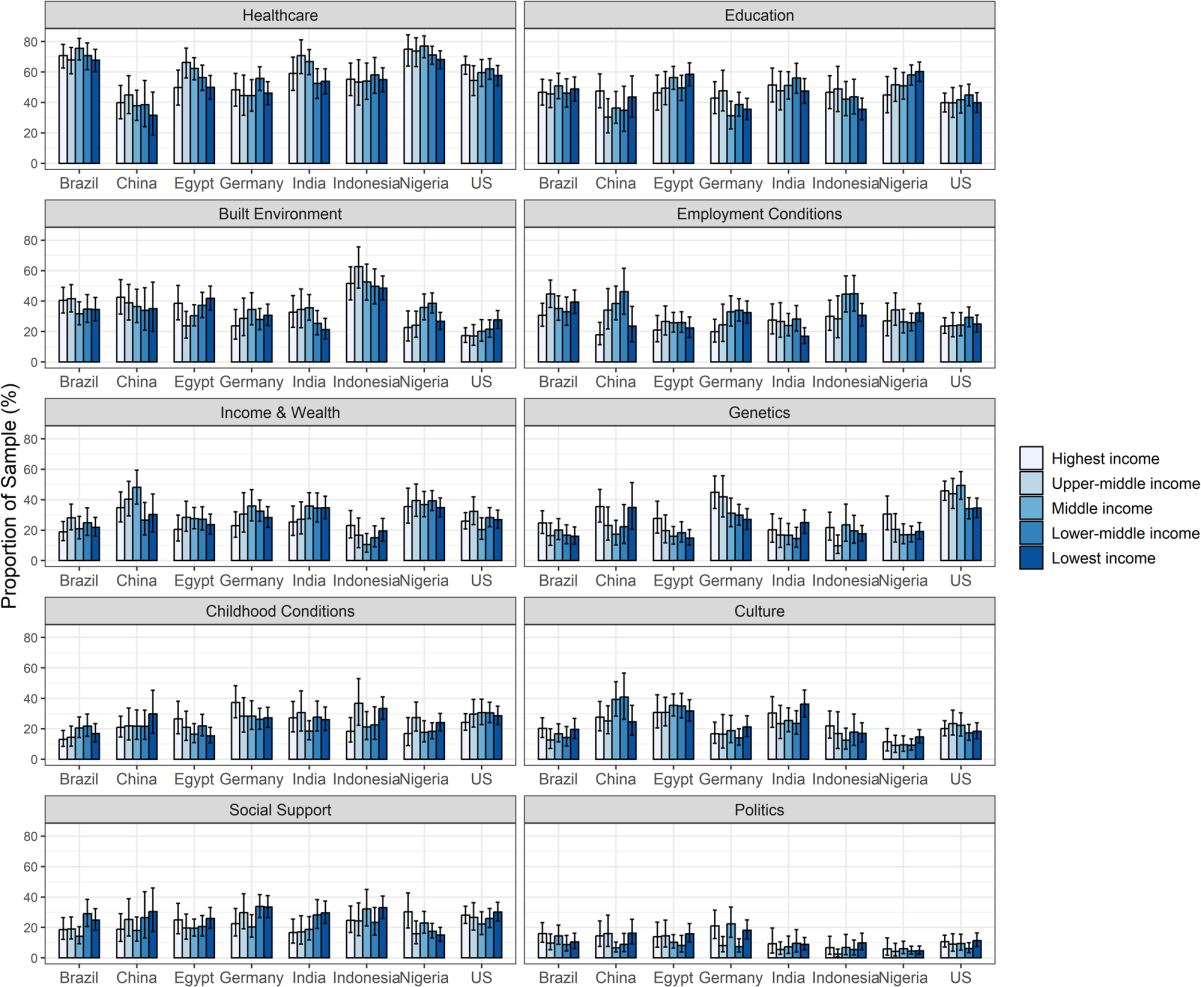
Rankings of what respondents think matters for health, stratified by country and income level. All responses with missing data for income levels (1123) were excluded (*N* = 7630). Results were weighted to adjust for age and gender. The rankings reflect a composite of the first, second, or third choice for important health determinants for health. Error bars represent the 95% confidence interval.

However, there were some notable exceptions. Respondents in the highest income group ranked genetics as the most important determinant of health in a higher proportion (32.4%, 95% CI: 29.0%, 35.8%) than the other income groups. While there was a general trend in within country analyses with higher income groups ranking genetics in higher proportions, the results varied slightly. In Brazil, China, Egypt, Germany, and Nigeria, the highest income group ranked genetics in higher proportions than other income groups. Conversely, in India, respondents in the lowest income group rated genetics in higher proportions than other income groups (Fig. 2). The analysis that assessed only the first choice showed comparable results with a few exceptions. (Supplementary Figs. 1 and 2).

Respondents in the lowest income group ranked social support as one of the three most important determinants of health in a higher proportion (27.9%, 95% CI: 25.3%, 30.7%) than the other income groups (Fig. 1). This general trend held true for the majority of countries; however, Nigeria was a notable exception with the highest income group reporting the highest proportion (30.3%, 95% CI: 19.7%, 42.6%) of respondents selecting social support as the most important determinant of health (Fig. 2).

The analysis looking at only the first choice generated more mixed results. The lowest income group reported the highest proportion in selecting social support in Germany (11.6%, 95% CI: 7.4%, 16.8%) and Indonesia (10.8%, 95% CI: 6.1%, 17.3%) compared to other income groups. The lower-middle income group reported the highest proportion selecting social support in China (10.9%, 95% CI: 2.5%, 27.2%) and India (11.2%, 95% CI: 4.5%, 21.7%) compared to other income groups. Conversely, the highest income group reported the highest proportions selecting social support in Egypt (7.2%, 95% CI: 2.0%, 16.9%), Nigeria (16.1%, 95% CI: 7.7%, 28.0%), and the United States (8.2%, 95% CI: 5.1%, 12.2%) compared to other income groups. In Brazil, the upper-middle income group reported the highest proportion selecting social support (6.0%, 95% CI: 3.1%, 10.2%) compared to other income groups. (Supplementary Fig. 2).

### Odds ratios of determinants of health rankings by income group

Table 2 shows the results of multivariable logistic regression models using the composite metric. Compared to the highest income group, all other groups—upper-middle income, middle income, lower-middle income and lowest income—had significantly lower odds of selecting genetics (OR = 0.69, 95% CI: [0.53, 0.89], *p* = 0.005; OR = 0.68, 95% CI: [0.54, 0.87], *p* = 0.002; OR = 0.62, 95% CI: [0.48, 0.78], *p* < 0.001; OR = 0.70, 95% CI: [0.54, 0.89], *p* = 0.004; respectively). In the second model, while not statistically significant, all other income groups reported higher odds ratios of selecting healthcare compared to the highest income group. Similarly, in the third model, all income groups reported higher odds ratios of selecting income and wealth compared to the highest income group. The results were statistically significant for the middle income group (OR = 1.27, 95% CI: [1.01, 1.59], *p* = 0.037). In the fourth model, compared to the highest income group, the upper-middle income and middle income groups had lower odds while the lower-middle income and lowest income groups had higher odds of selecting social support than the highest income group; none of these results were statistically significant. The multivariable models using respondents’ first choice showed comparable results for the genetics model and different results for other models (Supplementary Table 3).

**Table 2:** Logistic regression models for ranking genetics, healthcare, income and wealth, or social support as one of the three most important determinants of health

	Model 1: Genetics		Model 2: Healthcare			Model 3: Income and wealth			Model 4: Social Support	
Characteristic	OR [95% CI]	*p* value	OR [95% CI]	*p* value		OR [95% CI]		*p* value	OR [95% CI]	*p* value
Country										
United States	Ref.		Ref.				Ref.		Ref.	
Brazil	0.39 [0.30, 0.50]	<0.001	1.54 [1.21, 1.95]		<0.001		0.74 [0.57, 0.95]	0.019	0.73 [0.56, 0.95]	0.021
China	0.57 [0.41, 0.78]	<0.001	0.41 [0.31, 0.53]		<0.001		1.50 [1.14, 1.98]	0.004	0.85 [0.61, 1.19]	0.337
Egypt	0.40 [0.31, 0.52]	<0.001	0.83 [0.66, 1.04]		0.105		0.81 [0.63, 1.04]	0.102	0.83 [0.64, 1.07]	0.141
Germany	0.75 [0.59, 0.94]	0.012	0.62 [0.50, 0.78]		<0.001		1.21 [0.95, 1.53]	0.119	1.05 [0.83, 1.34]	0.661
India	0.40 [0.31, 0.54]	<0.001	0.95 [0.75, 1.20]		0.673		1.17 [0.91, 1.51]	0.225	0.86 [0.65, 1.13]	0.265
Indonesia	0.37 [0.27, 0.49]	<0.001	0.76 [0.59, 0.99]		0.041		0.56 [0.41, 0.76]	<0.001	1.09 [0.83, 1.43]	0.533
Nigeria	0.46 [0.36, 0.60]	<0.001	1.58 [1.26, 1.99]		<0.001		1.36 [1.08, 1.72]	0.010	0.69 [0.53, 0.90]	0.006
Gender										
Woman	Ref.		Ref.				Ref.		Ref.	
Man	0.90 [0.77, 1.04]	0.155	0.84 [0.74, 0.96]		0.010		1.28 [1.11, 1.47]	<0.001	0.93 [0.81, 1.08]	0.371
Age (years)										
18−34	Ref.		Ref.				Ref.		Ref.	
35−49	1.17 [1.00, 1.38]	0.055	1.09 [0.94, 1.25]		0.251		0.93 [0.80, 1.08]	0.325	0.93 [0.79, 1.09]	0.384
50−64	1.22 [0.96, 1.54]	0.100	1.11 [0.90, 1.39]		0.328		0.89 [0.70, 1.13]	0.331	1.09 [0.86, 1.37]	0.492
65 and older	1.78 [1.38, 2.31]	<0.001	0.86 [0.67, 1.10]		0.223		0.76 [0.57, 1.01]	0.062	1.06 [0.79, 1.41]	0.711
Income group										
Highest income	Ref.		Ref.				Ref.		Ref.	
Upper-middle income	0.69 [0.53, 0.89]	0.005	1.08 [0.85, 1.38]		0.512		1.27 [1.00, 1.61]	0.051	0.95 [0.73, 1.26]	0.738
Middle income	0.68 [0.54, 0.87]	0.002	1.12 [0.90, 1.39]		0.299		1.27 [1.01, 1.59]	0.037	0.84 [0.66, 1.07]	0.161
Lower-middle income	0.62 [0.48, 0.78]	<0.001	1.08 [0.88, 1.34]		0.456		1.24 [1.00, 1.53]	0.051	1.06 [0.84, 1.34]	0.638
Lowest income	0.70 [0.54, 0.89]	0.004	1.04 [0.84, 1.28]		0.733		1.16 [0.93, 1.44]	0.179	1.09 [0.86, 1.36]	0.480
Education										
College/university degree or more	Ref.		Ref.				Ref.		Ref.	
Secondary school	0.97 [0.80, 1.17]	0.728	1.11 [0.94, 1.30]		0.209		0.86 [0.73, 1.02]	0.091	1.21 [1.01, 1.44]	0.037
Less than secondary school/no formal education	0.95 [0.79, 1.16]	0.635	0.56 [0.47, 0.66]		<0.001		0.87 [0.74, 1.04]	0.121	1.52 [1.26, 1.84]	<0.001
Area of residence by urbanicity										
Urban	Ref.		Ref.				Ref.		Ref.	
Non-Urban	1.06 [0.91, 1.24]	0.474	0.86 [0.76, 0.99]		0.032		0.84 [0.73, 0.97]	0.017	1.20 [1.03, 1.39]	0.018

Responses with missing data on income (1123), education (787), and urban status (445) were removed (*N* = 7630). Predictors for both models were demographic variables and country. The rankings reflect a composite of the first, second, or third choice for important health determinants for health. Results were weighted to adjust for age and gender.

### Ranking of determinants by urbanicity status

**Fig. 3:**
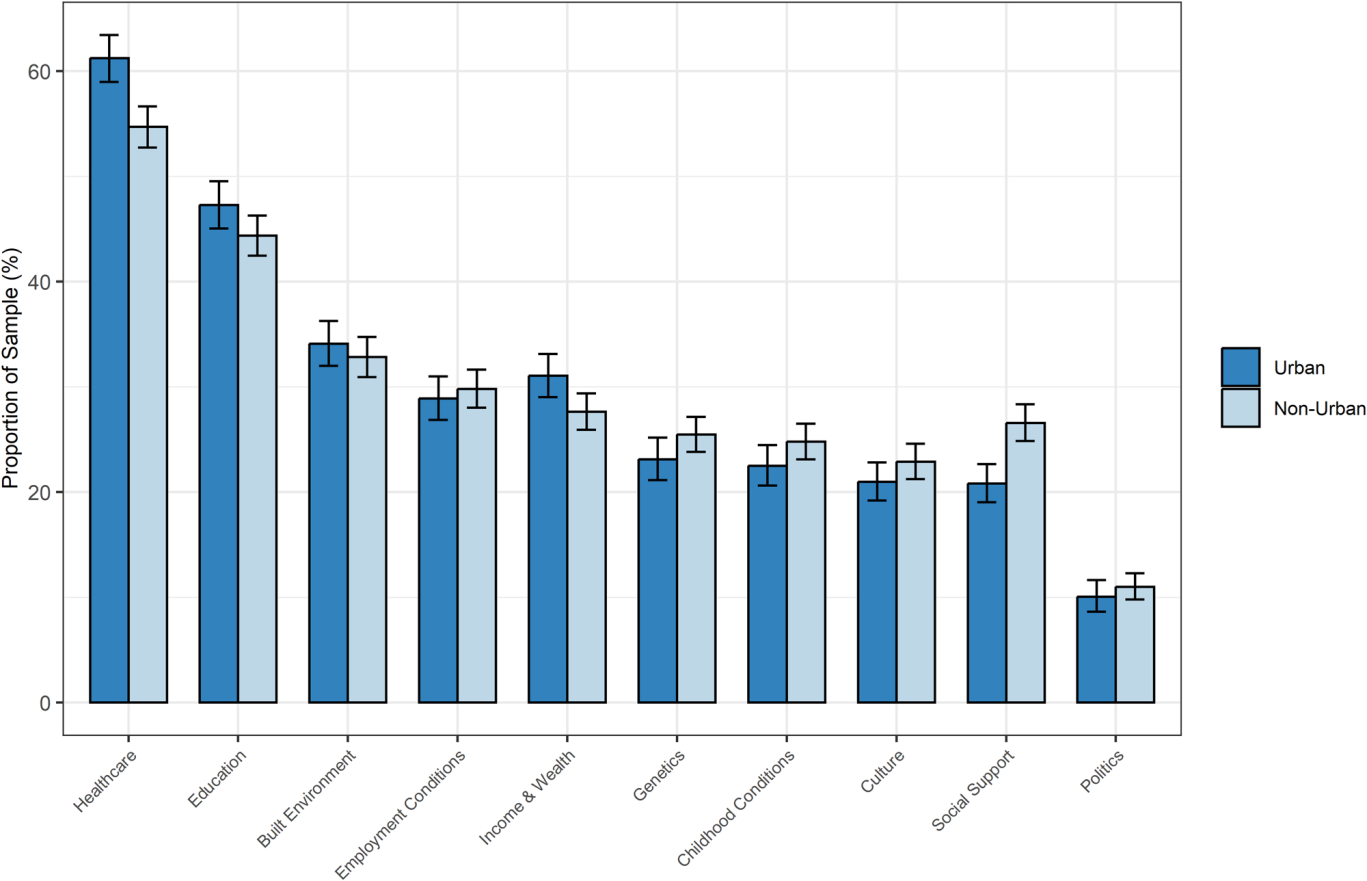
Rankings of what respondents think matters for health, stratified by urbanicity. All responses with missing data for urbanicity (445) were excluded (*N* = 8308). Results were weighted to adjust for age and gender. The rankings reflect a composite of the first, second, or third choice for important health determinants for health. Error bars represent the 95% confidence interval.

**Fig. 4:**
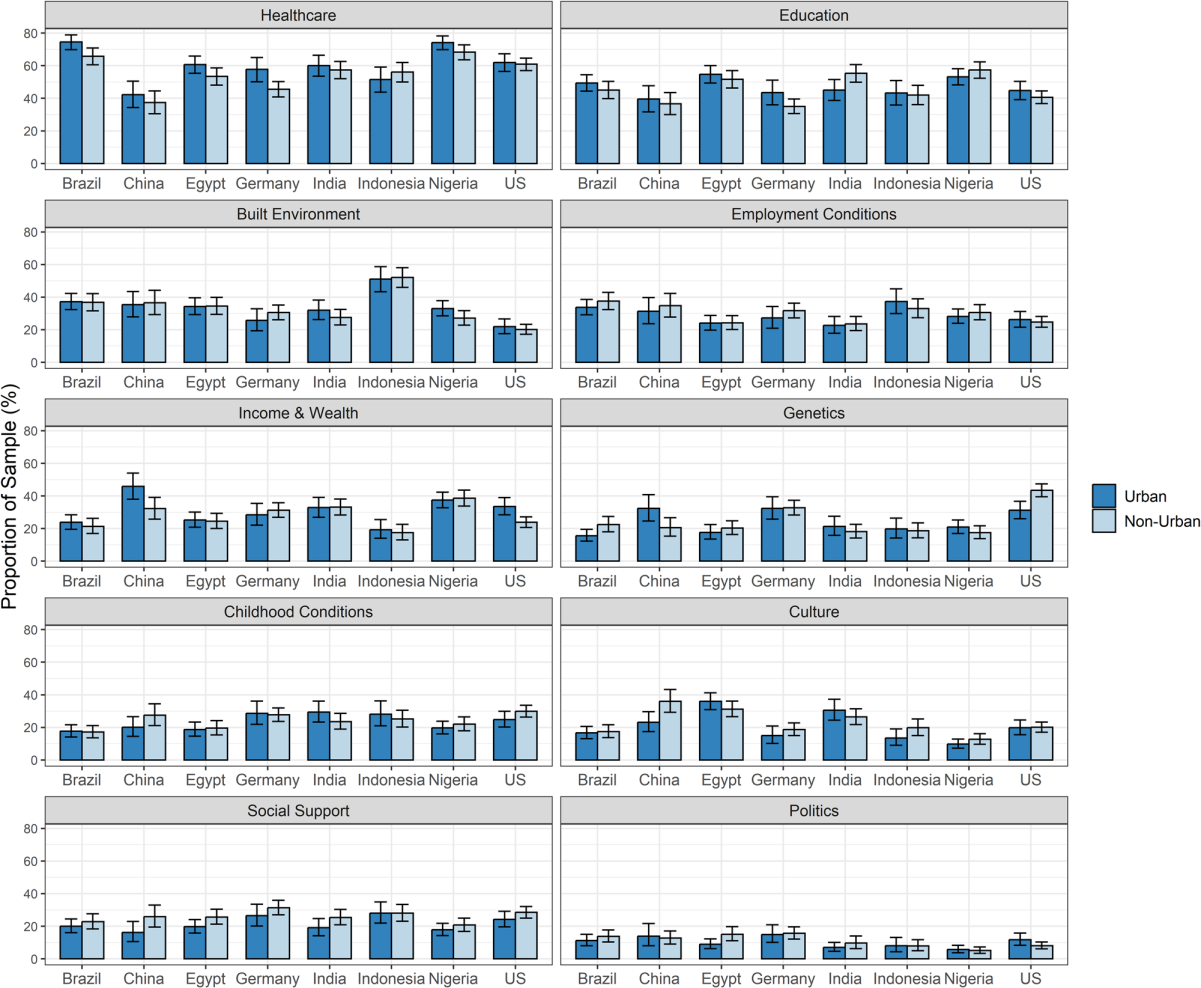
Rankings of what respondents think matters for health, stratified by country and urbanicity. All responses with missing data for urbanicity (445) were excluded (*N* = 8308). Results were weighted to adjust for age and gender. The rankings reflect a composite of the first, second, or third choice for important health determinants of health. Error bars represent the 95% confidence interval.

Figure 3 shows that, using the composite metric, respondents living in urban settings ranked healthcare, education, built environment, and income and wealth in higher proportions as important determinants of health compared to respondents living in non-urban settings. The opposite was true for employment conditions, genetics, childhood conditions, culture, social support, and politics. Associations with urbanicity were statistically significant for healthcare (*p* < 0.001), income and wealth (*p* = 0.012), and social support (*p* < 0.001). The largest absolute difference between the two groups was in ranking healthcare: 54.7% (95% CI: 52.7%, 56.6%) among non-urban respondents compared to 61.2% (95% CI: 59.0%, 63.4%) among urban respondents. The second largest difference was in ranking social support: 26.6% (95% CI: 24.9%, 28.3%) of non-urban respondents selected social support as the most important determinant of health compared to 20.8% (95% CI: 19.0%, 22.7%) in the urban group. These trends were consistent across countries, with the exception of Indonesia. (Fig. 4).

The results were comparable using respondents’ first choice except for built environment, income and wealth, and politics (Supplementary Fig. 3). Healthcare (*p* = 0.009) and social support (*p* = 0.004) had statistically significant associations with urbanicity. Within-country analysis results were generally comparable, with the exception of the ranking of genetics in Egypt. (Supplementary Fig. 4).

### Odds ratios of determinants of health rankings by urbanicity status

Table 2 shows the results of multivariable regression models using the composite metric. In their respective models, the non-urban group had significantly lower odds of selecting healthcare (OR = 0.86, 95% CI: [0.76, 0.99], *p* = 0.032) and income and wealth (OR = 0.84, 95% CI: [0.73, 0.97], *p* = 0.017). compared to the urban group. Conversely, the non-urban group had higher odds of selecting the social support (OR = 1.20, 95% CI: [1.03, 1.39], *p* = 0.018) compared to the urban group. The non-urban group had higher, but not statistically significant, odds of selecting the genetics compared to the urban group.

The multivariable models using respondents’ first choice showed different results for all models. The non-urban group showed higher odds of selecting genetics, income and wealth, and social support as well as lower odds of selecting healthcare compared to the urban group. None of the results were statistically significant. (Supplementary Table 3).

## Discussion

This analysis across eight countries demonstrated that, overall, respondents in the highest income group ranked genetics as one of the three most important determinants of health more frequently than other income groups. Conversely, respondents in lowest income group ranked social support as one of the most important determinants of health more frequently than other income groups. Moreover, we found notable variations in rankings of what matters for health based on the urbanicity status of respondents, particularly on the importance of healthcare and social support for health. A higher proportion of respondents living in urban settings ranked healthcare as an important determinant of health while a higher proportion of respondents in non-urban settings ranked social support as an important determinant of health.

Our findings showed that persons in the highest income group ranked genetics as an important determinant of heath substantially more often than did persons in all other income groups. This is consistent with results from previous country-level analyses^17,30^. These results may be explained by the observation that persons from a higher socioeconomic status being more likely to attribute successes, including better health outcomes, to their unique genetics and disposition^34,35,36^. Moreover, the wealthy within a country often have greater educational access and exposure to genetic concepts and, thus, the differences by income level may be due to information bias^37,38^. This can potentially lead to a bias towards overstating the role of genetics in shaping health outcomes among those from higher income groups.

We also showed that the lowest income group ranked social support as an important determinant of health at a substantially higher frequency than all other income groups. This is comparable with other studies that found lower income groups more likely to consider social factors as having a very strong effect on health^28^. This may reflect the fact that social support networks play an important role in lower income communities, sometimes serving as informal networks for health advice, coping tools, and sources for needed resources^39–41^.

We found a number of differences in ranking of health determinants by urbanicity status across countries, particularly on the importance of social support and healthcare. The rural-urban divide in perceptions on a wide range of issues is documented in the political science literature^32,42–44^. There is also evidence of an urban-rural divide in health literacy and perceptions around a number of diseases in the United States^45–47^. The prominence of social support as an important determinant of health for non-urban respondents may be explained by the greater salience of relational networks in rural communities. Research has shown the importance of social support networks as foundations of physical and emotional wellbeing in rural settings^48^. There is also some evidence of social support networks operating as informal sources of information and resources in rural communities where proper resources are scarce^49^. Conversely, the higher ranking of healthcare as an important determinant of health among respondents living in urban settings could be due to higher exposure to healthcare systems.

There are several limitations to this analysis. First, selection bias is inherent in the design—similar to all internet-based surveys—which can lead to results that are not representative of the populations within the countries surveyed. However, as shown in Table 1, the distribution of respondents across demographic factors was comparable across countries and to expectation. Second, while comparable to other surveys using this technology, the study had a high drop-out rate despite the use of various measures to maximize engagement and retain online respondents. Third, the list of determinants provided to respondents is based on the factors generally most cited in the literature on determinants of health and is by no means exhaustive. This analysis was meant to add to the scarce literature on the subject and provide representation across countries that differ around a number of axes. Future research should invest in representative survey methods that also yield a higher response rate.

Notwithstanding these limitations, our analysis highlights the fundamental observation that, regardless of the country, social and economic position within a country can shape perceptions of what matters for health. This showcases the need for investment in tailored communication efforts on the importance of SDoH that target different demographic groups.

### Supplementary information

Supplementary Information

Description of Additional Supplementary Files

Supplementary Data 1

Reporting Summary

## Data Availability

Source data for the figures are available as Supplementary Data 1. Other de-identified data are available on request to the corresponding author.
