# Supplementary material for: Perceptions of the determinants of health across income and urbanicity levels in eight countries
Source: Commun Med (Lond). 2024 Jun 6;4:107. doi: 10.1038/s43856-024-00493-z (PMC11156846; doi:10.1038/s43856-024-00493-z)
Supplement: Supplementary file 1 — Supplementary Information [file 43856_2024_493_MOESM1_ESM.pdf]

Supplementary Information

Supplementary Table 1. Monthly income category conversions for each country

Income category	Brazil (R$)	China (¥)	Egypt (£E)	Germany (€)	India (₹)	Indonesia (Rp)	Nigeria (₦)	US ($)
Lowest income	< 600	< 1500	< 700	< 1500	< 5000	< 1 million	< 5000	< 1800
Lower-middle income	601 - 1100	1500 - 3500	700 - 1500	1500 - 3500	5000 - 10000	1 - 2 million	5000 - 30000	1800 - 4000
Middle income	1101 - 1800	3501 - 8500	1501 - 3500	3501 - 5000	10001 - 50000	2 - 3 million	30001 - 100000	4001 - 6000
Upper-middle income	1801 - 3500	8501 - 16000	3501 - 8500	5001- 6500	50001 - 100000	3 - 5 million	100001- 350000	6001 - 10000
Highest income	> 3500	> 16000	> 8500	> 6500	> 100000	>5 million	> 350000	> 10000

Supplementary Table 2: Unweighted demographic characteristics of the study sample.

	Overall, N = 8,753	Brazil, N = 1,075	China, N = 1,282	Egypt, N = 1,082	Germany, N = 1,036	India, N =1,173	Indonesia, N = 1,026	Nigeria, N = 1,014	United States, N = 1,065
**Gender**									
Female	2,783 (31.8%)	411 (38.2%)	239 (18.6%)	250 (23.1%)	379 (36.6%)	353 (30.1%)	365 (35.6%)	302 (29.8%)	484 (45.4%)
Male	5,970 (68.2%)	664 (61.8%)	1,043 (81.4%)	832 (76.9%)	657 (63.4%)	820 (69.9%)	661 (64.4%)	712 (70.2%)	581 (54.6%)
**Age Group**									
18-24	3,062 (35.0%)	340 (31.6%)	464 (36.2%)	365 (33.7%)	199 (19.2%)	548 (46.7%)	466 (45.4%)	476 (46.9%)	204 (19.2%)
25-34	2,535 (29.0%)	296 (27.5%)	489 (38.1%)	352 (32.5%)	219 (21.1%)	383 (32.7%)	300 (29.2%)	312 (30.8%)	184 (17.3%)
35-44	1,453 (16.6%)	227 (21.1%)	194 (15.1%)	205 (18.9%)	235 (22.7%)	122 (10.4%)	151 (14.7%)	138 (13.6%)	181 (17.0%)
45-54	770 (8.8%)	120 (11.2%)	82 (6.4%)	85 (7.9%)	158 (15.3%)	54 (4.6%)	57 (5.6%)	60 (5.9%)	154 (14.5%)
55-64	462 (5.3%)	59 (5.5%)	14 (1.1%)	35 (3.2%)	133 (12.8%)	21 (1.8%)	24 (2.3%)	15 (1.5%)	161 (15.1%)
65 and older	471 (5.4%)	33 (3.1%)	39 (3.0%)	40 (3.7%)	92 (8.9%)	45 (3.8%)	28 (2.7%)	13 (1.3%)	181 (17.0%)
**Area of living**									
Large city	3,500 (42.1%)	539 (53.5%)	565 (47.2%)	512 (50.1%)	288 (29.2%)	417 (38.5%)	332 (33.6%)	507 (51.3%)	340 (32.9%)
Suburb	1,451 (17.5%)	116 (11.5%)	221 (18.4%)	111 (10.9%)	241 (24.4%)	162 (15.0%)	168 (17.0%)	114 (11.5%)	318 (30.8%)
Small town/village	2,280 (27.4%)	256 (25.4%)	287 (24.0%)	253 (24.8%)	325 (32.9%)	348 (32.1%)	316 (32.0%)	270 (27.3%)	225 (21.8%)
Rural area/farm	1,077 (13.0%)	96 (9.5%)	125 (10.4%)	146 (14.3%)	133 (13.5%)	156 (14.4%)	172 (17.4%)	98 (9.9%)	151 (14.6%)
**Income**									
Rich	1,404 (18.4%)	183 (19.9%)	302 (27.7%)	119 (12.7%)	141 (15.4%)	138 (14.3%)	158 (17.4%)	88 (9.6%)	275 (28.3%)

High income	999 (13.1%)	169 (18.4%)	187 (17.2%)	126 (13.4%)	90 (9.8%)	103 (10.7%)	111 (12.2%)	102 (11.1%)	111 (11.4%)
Middle income	1,524 (20.0%)	186 (20.2%)	287 (26.3%)	253 (26.9%)	141 (15.4%)	224 (23.3%)	142 (15.6%)	160 (17.4%)	131 (13.5%)
Low income	1,588 (20.8%)	162 (17.6%)	134 (12.3%)	205 (21.8%)	256 (27.9%)	204 (21.2%)	146 (16.0%)	269 (29.3%)	212 (21.8%)
Poor	2,115 (27.7%)	220 (23.9%)	180 (16.5%)	237 (25.2%)	290 (31.6%)	293 (30.5%)	353 (38.8%)	298 (32.5%)	244 (25.1%)
**Educational attainment**									
College/university degree or more	3,361 (42.2%)	233 (24.3%)	678 (59.7%)	482 (48.8%)	244 (25.5%)	536 (52.7%)	331 (34.6%)	418 (44.1%)	439 (43.8%)
Secondary school	2,246 (28.2%)	342 (35.6%)	165 (14.5%)	203 (20.5%)	382 (39.9%)	176 (17.3%)	390 (40.7%)	303 (32.0%)	285 (28.4%)
Less than secondary school/no formal education	2,359 (29.6%)	385 (40.1%)	293 (25.8%)	303 (30.7%)	331 (34.6%)	305 (30.0%)	237 (24.7%)	226 (23.9%)	279 (27.8%)

There are missing data on area of living (445), income (1123), and education (787).

Supplementary Table 3. Logistic regression models for ranking genetics, healthcare, income and wealth, or social support as the most important determinant of health

	Model 1: Genetics		Model 2: Healthcare		Model 3: Income and wealth		Model 4: Social Support	
Characteristic	OR [95% CI]	p-value	OR [95% CI]	p-value	OR [95% CI]	p-value	OR [95% CI]	p-value
Country								
United States	Ref		Ref		Ref		Ref	
Brazil	0.39 [0.25, 0.61]	<0.001	1.57 [1.25, 1.97]	<0.001	0.49 [0.30, 0.79]	0.003	0.54 [0.34, 0.86]	0.009
China	0.66 [0.42, 1.04]	0.074	0.40 [0.28, 0.59]	<0.001	1.69 [1.17, 2.46]	0.006	0.98 [0.56, 1.69]	0.934
Egypt	0.44 [0.29, 0.66]	<0.001	0.74 [0.57, 0.95]	0.019	0.70 [0.46, 1.07]	0.101	0.64 [0.38, 1.08]	0.093
Germany	0.93 [0.67, 1.29]	0.643	0.68 [0.52, 0.88]	0.003	0.81 [0.55, 1.18]	0.265	1.23 [0.84, 1.82]	0.290
India	0.42 [0.26, 0.68]	<0.001	1.01 [0.78, 1.31]	0.949	0.76 [0.51, 1.12]	0.169	1.17 [0.72, 1.90]	0.534
Indonesia	0.37 [0.24, 0.58]	<0.001	0.56 [0.41, 0.76]	<0.001	0.46 [0.27, 0.78]	0.004	1.14 [0.72, 1.81]	0.563
Nigeria	0.47 [0.32, 0.71]	<0.001	1.34 [1.06, 1.70]	0.014	0.75 [0.51, 1.10]	0.145	0.94 [0.59, 1.50]	0.795
Gender								
Women	Ref		Ref		Ref		Ref	
Men	0.82 [0.66, 1.03]	0.091	0.83 [0.72, 0.96]	0.011	1.95 [1.52, 2.49]	<0.001	1.05 [0.80, 1.36]	0.743
Age (years)								
18-34	Ref		Ref		Ref		Ref	
35-49	1.24 [0.96, 1.61]	0.099	1.15 [0.99, 1.34]	0.073	1.12 [0.88, 1.42]	0.375	1.11 [0.83, 1.48]	0.477
50-64	1.18 [0.86, 1.62]	0.318	1.11 [0.88, 1.39]	0.389	0.90 [0.62, 1.29]	0.567	0.84 [0.55, 1.29]	0.431
65 and older	1.80 [1.25, 2.61]	0.002	0.81 [0.61, 1.09]	0.171	0.65 [0.41, 1.03]	0.064	1.59 [1.04, 2.44]	0.034

**Income**								
Highest income	Ref		Ref		Ref		Ref	
Upper-middle income	0.68 [0.46, 1.01]	0.055	1.03 [0.80, 1.33]	0.836	0.91 [0.60, 1.37]	0.644	0.70 [0.46, 1.06]	0.093
Middle income	0.67 [0.47, 0.96]	0.028	1.21 [0.96, 1.53]	0.110	0.90 [0.64, 1.29]	0.577	0.65 [0.41, 1.03]	0.065
Lower-middle income	0.62 [0.44, 0.87]	0.006	1.02 [0.81, 1.28]	0.872	1.17 [0.83, 1.64]	0.362	0.91 [0.60, 1.37]	0.650
Lowest income	0.63 [0.44, 0.89]	0.010	0.90 [0.71, 1.14]	0.374	1.14 [0.81, 1.59]	0.459	0.91 [0.62, 1.32]	0.607
**Education**								
College/university degree or more	Ref		Ref		Ref		Ref	
Secondary school	0.73 [0.56, 0.97]	0.031	1.13 [0.96, 1.34]	0.135	0.81 [0.61, 1.06]	0.127	1.35 [1.01, 1.80]	0.042
Less than secondary school/no formal education	0.91 [0.68, 1.22]	0.516	0.84 [0.69, 1.01]	0.066	0.88 [0.67, 1.15]	0.339	1.98 [1.44, 2.74]	<0.001
**Area of residence by urbanicity**								
Urban	Ref		Ref		Ref		Ref	
Non-Urban	1.18 [0.93, 1.50]	0.170	0.92 [0.80, 1.06]	0.266	1.01 [0.80, 1.28]	0.906	1.17 [0.91, 1.52]	0.221

Responses with missing data on income (1123), education (787), and urban status (445) were removed. Predictors for both models were demographic variables and country. Rankings reflect a respondent's first choice. Results were weighted to adjust for age and gender.

Supplementary Figure 1. Rankings of what respondents think matters for health, stratified by income level

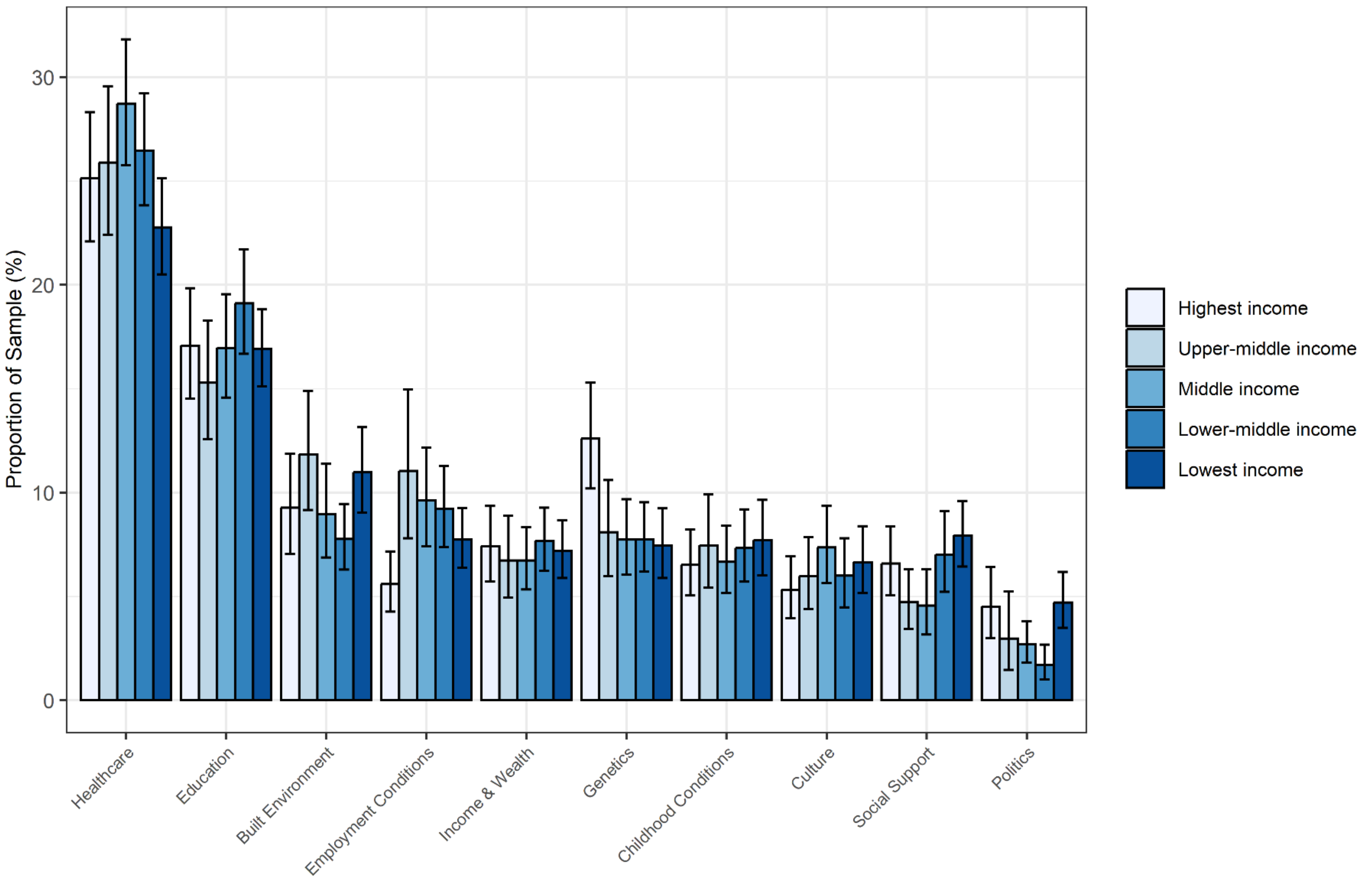

All responses with missing data for income levels (1123) were excluded. Results were weighted to adjust for age and gender. Rankings reflect a respondent's first choice.

Supplementary Figure 2. Rankings of what respondents think matters for health, stratified by country and income level

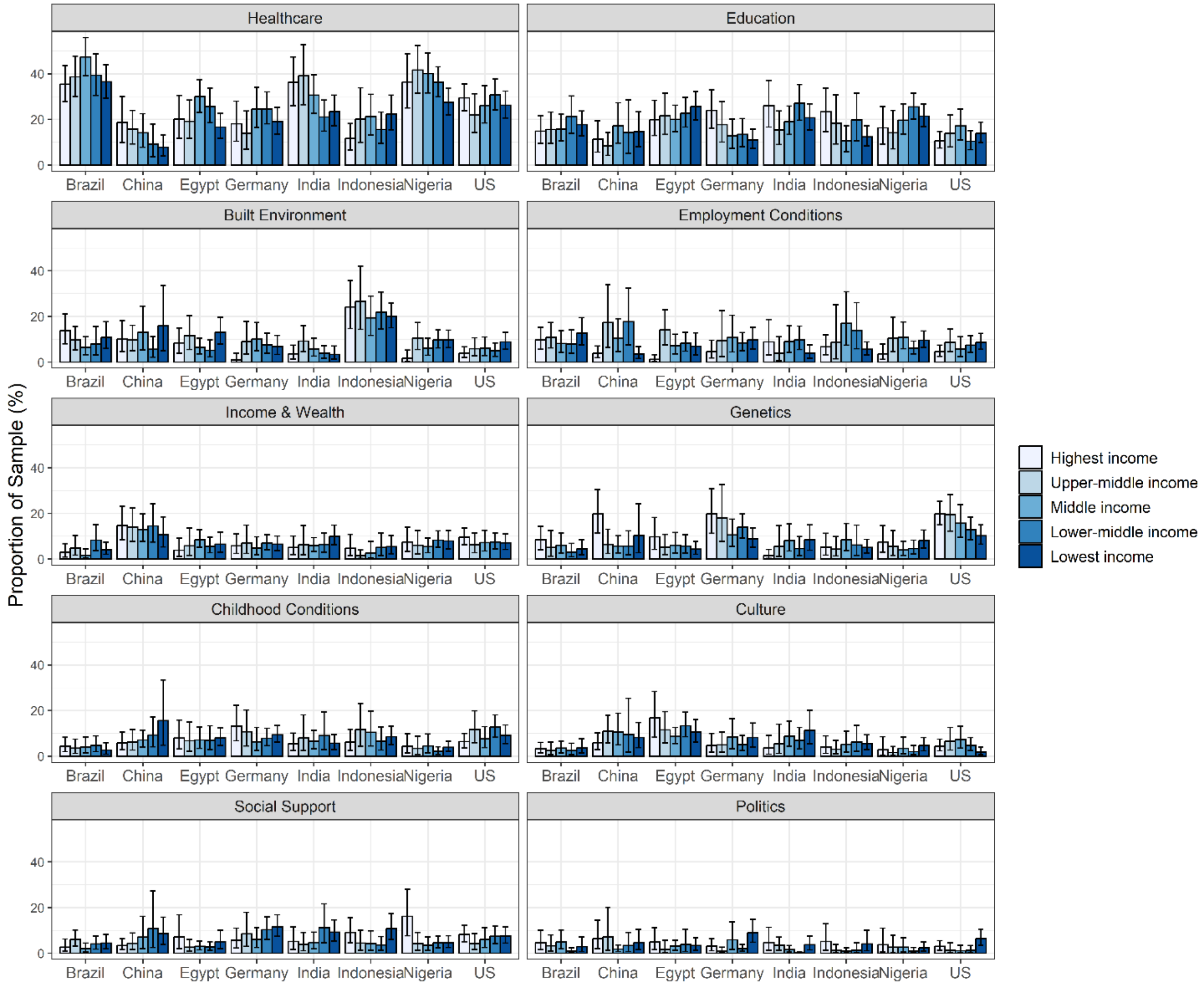

All responses with missing data for income levels (1123) were excluded. Results were weighted to adjust for age and gender. Rankings reflect a respondent's first choice.

Supplementary Figure 3. Rankings of what respondents think matters for health, stratified by urbanicity

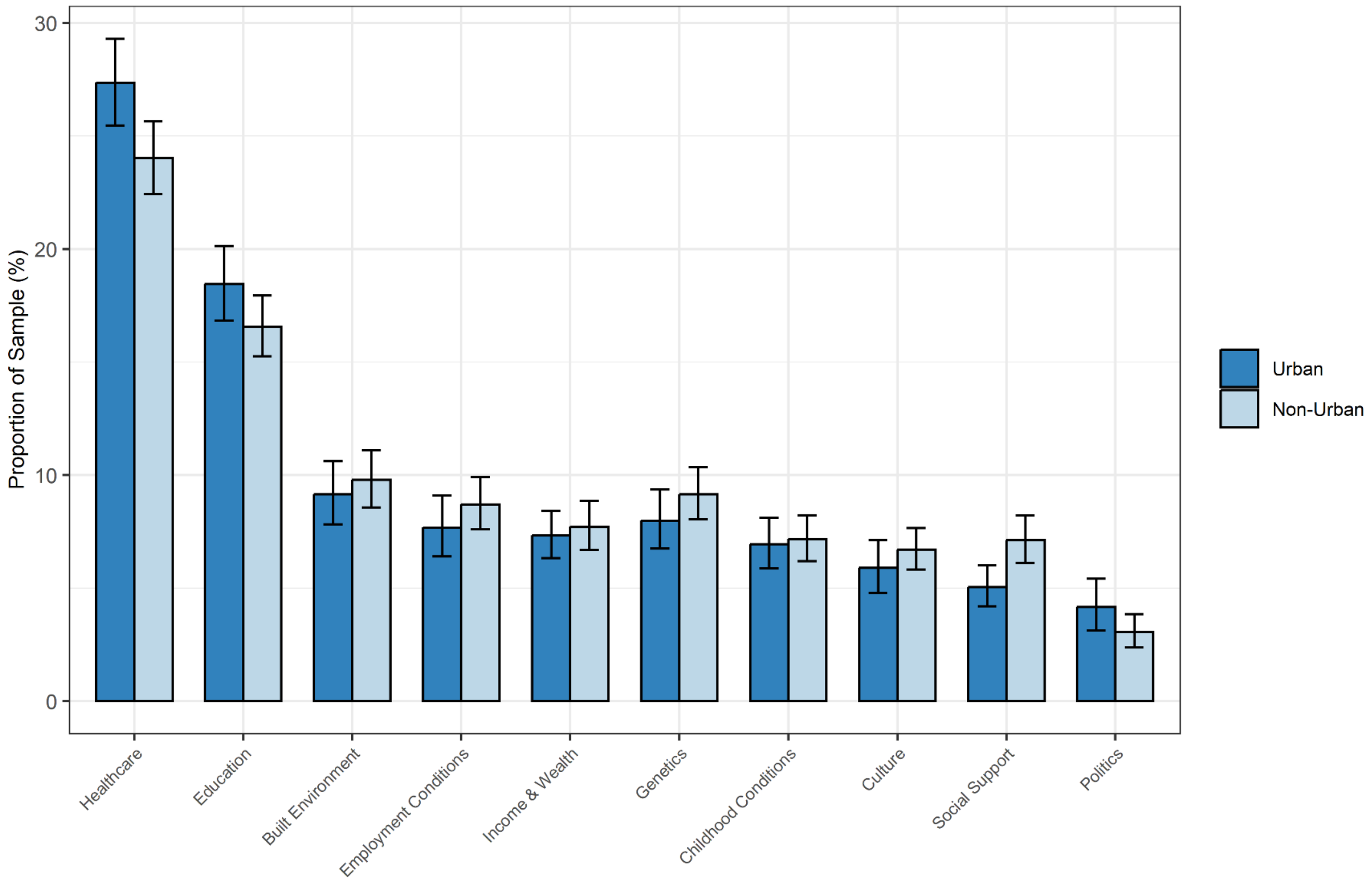

All responses with missing data for urbanicity (445) were excluded. Results were weighted to adjust for age and gender. The rankings reflect a composite of the first, second, or third choice for important health determinants for health. Rankings reflect a respondent's first choice.

Supplementary Figure 4. Rankings of what respondents think matters for health, stratified by country and urbanicity

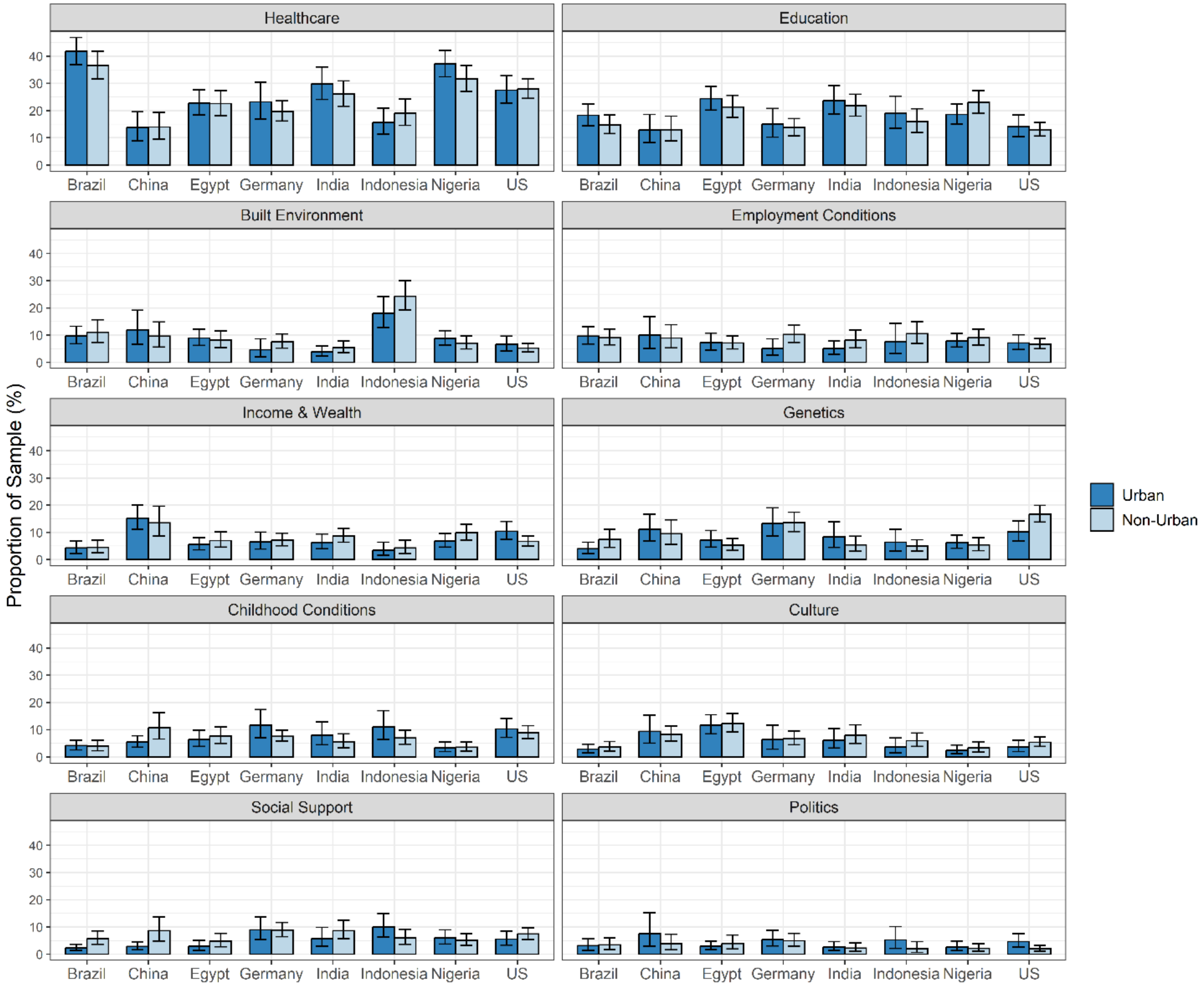

All responses with missing data for urbanicity (445) were excluded. Results were weighted to adjust for age and gender. The rankings reflect a composite of the first, second, or third choice for important health determinants for health. Rankings reflect a respondent's first choice.
