# Supplementary material for: Perceptions of the determinants of health across income and urbanicity levels in eight countries
Source: Commun Med (Lond). 2024 Jun 6;4:107. doi: 10.1038/s43856-024-00493-z (PMC11156846; doi:10.1038/s43856-024-00493-z)
Supplement: Supplementary file 2 — Description of Additional Supplementary Files [file 43856_2024_493_MOESM2_ESM.pdf]

Description of Additional Supplementary Files

File name: Supplementary Data 1
Description: Source data
