# Supplementary material for: Perceptions of the determinants of health across income and urbanicity levels in eight countries
Source: Commun Med (Lond). 2024 Jun 6;4:107. doi: 10.1038/s43856-024-00493-z (PMC11156846; doi:10.1038/s43856-024-00493-z)
Supplement: Supplementary file 4 — Reporting Summary [file 43856_2024_493_MOESM4_ESM.pdf]

nature portfolio

Corresponding author(s): COMMSMED-23-0295-T

Last updated by author(s): Mar 21, 2024

Reporting Summary

Nature Portfolio wishes to improve the reproducibility of the work that we publish. This form provides structure for consistency and transparency in reporting. For further information on Nature Portfolio policies, see our Editorial Policies and the Editorial Policy Checklist.

Statistics

For all statistical analyses, confirm that the following items are present in the figure legend, table legend, main text, or Methods section.

n/a	Confirmed	
☐	☒	The exact sample size (*n*) for each experimental group/condition, given as a discrete number and unit of measurement
☐	☒	A statement on whether measurements were taken from distinct samples or whether the same sample was measured repeatedly
☐	☒	The statistical test(s) used AND whether they are one- or two-sided<br>*Only common tests should be described solely by name; describe more complex techniques in the Methods section.*
☐	☒	A description of all covariates tested
☐	☒	A description of any assumptions or corrections, such as tests of normality and adjustment for multiple comparisons
☐	☒	A full description of the statistical parameters including central tendency (e.g. means) or other basic estimates (e.g. regression coefficient) AND variation (e.g. standard deviation) or associated estimates of uncertainty (e.g. confidence intervals)
☒	☐	For null hypothesis testing, the test statistic (e.g. *F*, *t*, *r*) with confidence intervals, effect sizes, degrees of freedom and *P* value noted<br>*Give P values as exact values whenever suitable.*
☒	☐	For Bayesian analysis, information on the choice of priors and Markov chain Monte Carlo settings
☒	☐	For hierarchical and complex designs, identification of the appropriate level for tests and full reporting of outcomes
☒	☐	Estimates of effect sizes (e.g. Cohen's *d*, Pearson's *r*), indicating how they were calculated

Our web collection on statistics for biologists contains articles on many of the points above.

Software and code

Policy information about availability of computer code

Data collection	RIWI collected data using randomized domain intercept technology (RDIT™). Users who made errors while typing website addresses in the URL bar were redirected to domains owned or controlled by RIWI. Using a series of algorithms, RIWI invited a random sample to participate in the survey. Respondents received the survey in a language predominantly used in their country. RDIT™ uses filters to prevent non-human respondents from responding.
Data analysis	Data analysis was conducted in R version 4.1.2, using the packages "tidyverse", "data.table", "survey", "srvyr", "gt", and "gtsummary" for cleaning and analysis. RIWI provided survey weights created from the age and gender of sample respondents. We used the "tidyverse" and "data.table" packages to clean the code and "survey", "srvyr", "gt", and "gtsummary" for bivariable and multivariable regression analyses in both tables and figures. We created custom functions for displaying the p-value to three digits.

For manuscripts utilizing custom algorithms or software that are central to the research but not yet described in published literature, software must be made available to editors and reviewers. We strongly encourage code deposition in a community repository (e.g. GitHub). See the Nature Portfolio guidelines for submitting code & software for further information.

Data

Policy information about availability of data

All manuscripts must include a data availability statement. This statement should provide the following information, where applicable:

- Accession codes, unique identifiers, or web links for publicly available datasets
- A description of any restrictions on data availability
- For clinical datasets or third party data, please ensure that the statement adheres to our policy

De-identifiable data are available upon reasonable request.

Research involving human participants, their data, or biological material

Policy information about studies with human participants or human data. See also policy information about sex, gender (identity/presentation), and sexual orientation and race, ethnicity and racism.

Reporting on sex and gender	Gender was determined based on self-reporting. There were 2,783 women and 5,970 men who completed the survey. Gender was used in the creation of weights.
Reporting on race, ethnicity, or other socially relevant groupings	Questions on race and ethnicity were not present in the survey. Data was collected on age, income quintile, education, and area of residence. Age was used in the creation of weights. Income quintile, education, and area of residence controlled for confounding variables in regression models.
Population characteristics	The unweighted age distribution of the population included 3,062 ages 18-24 years, 2,535 ages 25-34 years, 1,453 ages 35-44 years, 770 ages 45-54 years, 462 ages 55-64 years, and 471 ages 65 or older. The unweighted income distribution of the population included 1,404 highest income, 999 upper-middle income, 1,524 middle income, 1,588 lower-middle income, and 2,115 lowest income respondents. The unweighted education distribution consisted of 3,361 with a college or university degree or more, 2,246 with secondary school, and 2,359 with less than secondary school. The unweighted urbanicity distribution included 3,500 urban and 4,808 non-urban respondents.
Recruitment	Participants were recruited online via RIWI. Users who made errors while typing website addresses in the URL bar were redirected to domains owned or controlled by RIWI. Using a series of algorithms, RIWI invited a random sample to participate in the survey. Respondents received the survey in a language predominantly used in their country. RDIT™ uses filters to prevent non-human respondents from responding.
Ethics oversight	This research was exempted from a comprehensive ethical review by the Institutional Review Board (IRB) at Boston University (IRB Number: H-40806) due to data anonymity.

Note that full information on the approval of the study protocol must also be provided in the manuscript.

Field-specific reporting

Please select the one below that is the best fit for your research. If you are not sure, read the appropriate sections before making your selection.

☐ Life sciences ☒ Behavioural & social sciences ☐ Ecological, evolutionary & environmental sciences

For a reference copy of the document with all sections, see nature.com/documents/nr-reporting-summary-flat.pdf

Behavioural & social sciences study design

All studies must disclose on these points even when the disclosure is negative.

Study description	quantitative cross-sectional
Research sample	The research sample consisted of adult (age 18 years or older) Internet users in eight countries (Brazil, China, Egypt, Germany, India, Indonesia, Nigeria, and the United States) who use the address bar when typing. The sample is not representative: there were 2,783 women and 5,970 men who answered the full survey; weights were created using gender and age to match population distributions at the national level.
Sampling strategy	The sampling strategy was a random sample using the RDIT™ algorithms from RIWI. The sample size was determined based on discussions with RIWI and budget considerations, so the survey was provided in each country until 1000 complete responses were elicited.
Data collection	RIWI collected responses online.
Timing	Data collection occurred between September 16th, 2020 and November 1st, 2020.
Data exclusions	Participants with missing data on income (1123), education (787), and urban status (445) were excluded from the analyses.

Non-participation	The survey link response rate was 23.4%.
Randomization	Participants were randomly assigned to two survey question orders.

Reporting for specific materials, systems and methods

We require information from authors about some types of materials, experimental systems and methods used in many studies. Here, indicate whether each material, system or method listed is relevant to your study. If you are not sure if a list item applies to your research, read the appropriate section before selecting a response.

Materials & experimental systems

n/a	Involved in the study
☒	☐ Antibodies
☒	☐ Eukaryotic cell lines
☒	☐ Palaeontology and archaeology
☒	☐ Animals and other organisms
☒	☐ Clinical data
☒	☐ Dual use research of concern
☒	☐ Plants

Methods

n/a	Involved in the study
☒	☐ ChIP-seq
☒	☐ Flow cytometry
☒	☐ MRI-based neuroimaging
